# Genome Sequence of *Lactobacillus brevis* Strain D6, Isolated from Smoked Fresh Cheese

**DOI:** 10.1128/genomeA.00264-16

**Published:** 2016-04-07

**Authors:** Ravi Kant, Ksenija Uroić, Ulla Hynönen, Blaženka Kos, Jagoda Šušković, Airi Palva

**Affiliations:** a Department of Veterinary Biosciences, Faculty of Veterinary Medicine, University of Helsinki, Helsinki, Finland; b Faculty of Food Technology and Biotechnology, Laboratory of Antibiotic, Enzyme, Probiotic and Starter Culture Technologies, University of Zagreb, Zagreb, Croatia

## Abstract

The autochthonous *Lactobacillus brevis* strain D6, isolated from smoked fresh cheese, carries a 45-kDa S-layer protein. Strain D6 has shown adhesion to extracellular matrix proteins and to Caco-2 intestinal epithelial cells, as well as immunomodulatory potential and beneficial milk technological properties. Hence, it could be used as a potential probiotic starter culture for cheese production.

## GENOME ANNOUNCEMENT

An autochthonous *Lactobacillus brevis* strain, D6, was isolated from traditionally produced smoked fresh cheese originating from a specific Croatian ecological locality of the Zagorje region. These artisanal cheeses are produced by the spontaneous fermentation of cow’s milk and harbor a rich diversity of lactic acid bacteria (1). As they are manufactured from non-pasteurized milk without using commercial starter cultures, their sensory properties depend on the composition of lactic acid bacteria in raw milk, where the present bacteria act as natural starter cultures (2). *Lactobacillus brevis* is an abundant *Lactobacillus* species in traditionally produced smoked fresh cheeses. It is a microaerophilic, Gram-positive and obligatory hetero-fermentative lactic acid bacterium. It has been isolated from many different environments such as sauerkraut, sourdough, silage, and the intestinal tract of humans and animals, and it is involved in the production of a wide spectrum of fermented products (3, 4). For instance, as a hetero-fermentative bacterium, *L. brevis* plays a major role in the fermentation of Italian sweet doughs during the production of Panettone, Colomba, and Pandoro breads and various mini-cakes, where it is a predominant starter culture in the mixture of lactic acid bacteria used (5).

Some *L. brevis* strains with specific surface (S-layer) proteins possess a variety of functional properties that make them both potential probiotics and good vaccine vector candidates (6, 7). The strain *L. brevis* D6 carries a 45-kDa S-layer protein and has shown adhesion to extracellular matrix proteins and to Caco-2 intestinal epithelial cells, as well as immunomodulatory potential. Together with the putative protective role of S-layer proteins, suggested by the enhanced survival of S-layer carrying *L. brevis* D6 in rigorous conditions, such as those encountered in the gastrointestinal tract and during the process of freeze-drying, this strain could be defined as a potential probiotic starter culture for cheese production (8).

The genomic DNA of *L. brevis* D6 was extracted from 1 mL of overnight culture grown in MRS medium using the Wizard R Genomic DNA purification kit (Promega) and the entire genome was sequenced using the Roche 454 Life Sciences GS FLX system. The obtained sequences were assembled using Newbler, ending up with a 12× coverage of each genome. Annotation was performed based on the PGAAP analysis (http://www.ncbi.nlm.nih.gov/genomes/static/Annotation_pipeline_procedures.txt) and manual verification.

The draft genome of *L. brevis* D6 strain contains 2,591,675 nucleotides with an overall G+C content of 45.6% in 123 contigs. The chromosome contains 2,569 protein-coding sequences (CDS) with 76 RNA genes, 121 pseudo genes, and one clustered regularly interspaced short palindromic repeat (CRISPR) array. Using the SignalP software (9), 140 proteins in total were predicted to be secreted.

### Nucleotide sequence accession number.

The draft genome sequence of *Lactobacillus brevis* strain D6 is available in GenBank under the accession number LQNG00000000.
